# Inferring protein–protein interaction complexes from immunoprecipitation data

**DOI:** 10.1186/1756-0500-6-468

**Published:** 2013-11-15

**Authors:** Joachim Kutzera, Huub CJ Hoefsloot, Anna Malovannaya, August B Smit, Iven Van Mechelen, Age K Smilde

**Affiliations:** 1 Biosystems Data Analysis, Swammerdam Institute for Life Sciences, University of Amsterdam, Amsterdam, The Netherlands; 2 Netherlands Institute for Systems Biology, University of Amsterdam, Amsterdam, The Netherlands; 3 Department of Molecular and Cellular Neurobiology, Center for Neurogenomics and Cognitive Research, VU University Amsterdam, Amsterdam, The Netherlands; 4 Department of Molecular and Cellular Biology, Baylor College of Medicine, Houston, TX, 77030, USA; 5 Faculty of Psychology and Educational Sciences, Katholieke Universiteit Leuven, Leuven, Belgium

**Keywords:** Protein–protein interactions, Proteomics, Protein complexes, Immunoprecipitation

## Abstract

**Background:**

Protein–protein interactions in cells are widely explored using small–scale experiments. However, the search for protein complexes and their interactions in data from high throughput experiments such as immunoprecipitation is still a challenge. We present "4N", a novel method for detecting protein complexes in such data. Our method is a heuristic algorithm based on Near Neighbor Network (3N) clustering. It is written in R, it is faster than model-based methods, and has only a small number of tuning parameters. We explain the application of our new method to real immunoprecipitation results and two artificial datasets. We show that the method can infer protein complexes from protein immunoprecipitation datasets of different densities and sizes.

**Findings:**

4N was applied on the immunoprecipitation dataset that was presented by the authors of the original 3N in Cell 145:787–799, 2011. The test with our method shows that it can reproduce the original clustering results with fewer manually adapted parameters and, in addition, gives direct insight into the complex–complex interactions. We also tested 4N on the human "Tip49a/b" dataset. We conclude that 4N can handle the contaminants and can correctly infer complexes from this very dense dataset. Further tests were performed on two artificial datasets of different sizes. We proved that the method predicts the reference complexes in the two artificial datasets with high accuracy, even when the number of samples is reduced.

**Conclusions:**

4N has been implemented in R. We provide the sourcecode of 4N and a user-friendly toolbox including two example calculations. Biologists can use this 4N-toolbox even if they have a limited knowledge of R. There are only a few tuning parameters to set, and each of these parameters has a biological interpretation. The run times for medium scale datasets are in the order of minutes on a standard desktop PC. Large datasets can typically be analyzed within a few hours.

## Findings

### Background

Protein–protein interactions (PPIs) constitute the core of inner cell communication [1,2]. Large–scale analysis of PPIs in cells became possible due to the development of high throughput measurement methods, in particular, affinity immunoprecipitation followed by mass spectrometry (AP/IP–MS) [3-5]. During an IP experiment, an antibody (an immune system protein raised in a host species to recognize specific foreign target proteins) binds to its target antigen in the cell sample. The antigen and proteins that are bound can be effectively isolated from the sample via interaction with an antibody and quantified and identified directly by mass-spectrometry [3,6]. IP-experiments using various antibodies on the same sample result in different, but possibly partly identical sets of identified proteins that have different abundances in each experiment [7].

Protein complexes work as functional units in the interaction network, and the members of a complex do not appear individually. For this reason, proteins of the same complex are predicted to have a similar abundance in different IP–experiments. Consequently, a similarity-based cluster analysis of these datasets leads to clusters that represent the protein complexes. The clustering should be based on both parts of the information, namely, the occurrence of proteins in the samples and their relative abundance values. Complex–complex interactions (CCIs) as a coarser view on the PPI network can be represented by a clustering in which clusters are allowed to share proteins as proof of the interaction.

Large–scale IP/MS study by Gavin et al. first described such analysis for proteome wide characterization of protein complexes in yeast [3]. Subsequently, a variety of studies describing different methods to find PPI networks in this dataset emerged, notably including the work of Krogan [8], Ethan [9], Collins [10] and Xie [11]. Different methods were also presented and compared in [12].

A medium scale IP dataset for the components of the human Tip49a/b–PPI complex was presented by Sardiu et al. and used to illustrate complex inference [13]. Several clustering methods were compared on the same dataset in [14]. More recent methods which were applied to Tip49a/b are biclust [15] and bi-map [16]. Both of these methods are model based and both publications claim to outperform previous methods. Biclust is freely accessible and easy to handle. We have selected this method for the comparison with 4N for this reason.

The different clustering methods are scattered widely, and all studies concluded that there is no standard method available currently that is suitable for clustering all kinds of IP–based interaction data. In addition, some of those methods, for example biclust, involve many parameters and are very time–consuming as they need many iterations to get to a result.

Malovannaya et al. introduced a method called Near Neighbor Network clustering (3N) for predicting core protein complexes in their large–scale IP/MS study on human cells [17]. This algorithm has been implemented within their local proteomics database and has four external parameters that influence the result. The authors optimized these parameters manually to identify several complexes that are described in the literature as biologically relevant and proved that 3N can find them in their specific dataset.

In this study, we present a new universal complex inference method that is based on the idea of 3N clustering which we call "4N". Most of its parameters can be set automatically; moreover, it contains tools for visualizing complexes and their interactions. Major advantages of 4N over other methods include that it can process very large datasets, that it allows an immediate insight into the data and that it directly shows the effect of the parameters on the cluster result. 4N is simple and much faster than methods based on probabilistic models, such as biclust. The high speed of 4N in combination with the visualisation tools allows the quick interactive search for protein complexes. A software toolbox written in R is available in the software section on the website www.bdagroup.nl. It includes the algorithm itself, documentation, running examples and methods for plotting and evaluation of the cluster result based on given reference complexes.

The article starts with the description of the empirical and simulated IP datasets that we used for testing 4N. The 3N and 4N methods are described in detail thereafter. The article closes with detailed results for all datasets and a Discussion and conclusion section.

### Data description

#### Empirical datasets

For a direct comparison with 3N, we did an analysis using our methods on the very large dataset from the original 3N publication [18], which contains about 3200 IPs with 11500 identified gene products. We name this dataset "malovIP". To mimic a targeted IP/MS dataset of intermediate size, we applied 4N with a low *U* of 0.125 to "malovIP" and removed all proteins that were not assigned to near neighbor networks. The subset contained, besides many other proteins, all protein subunits of the Integrator [19], Mediator [20,21], and HDAC1/2 repressor complexes from the "malovIP" matrix. This derivative set is called "malovIP_subset" in our work. The reference clusters for both "malovIP" datasets were the 6 clusters described in Figure two(A,B) of [18], the "mediator"-complex in Figure three(B) and the 3 complexes in Figure four(A) of the same publication.

The next dataset that we analyzed is the "human Tip49a/b" from [13]. We obtained a version of this dataset that also includes proteins that are not in complexes from the Supplementary Material of [16]. Reference complexes for this dataset were taken from Figure three in [16]. A detailed list of the reference proteins can be found in our Additional file 1.

#### Simulated datasets

Unlike PPI networks from yeast–two–hybrid studies, only a few IP–based datasets are available; information about reference complexes especially is rare. We created another small–scale, and a medium–scale IP dataset from two simulated networks for this reason. The two PPI networks were created with help of the protein interaction database string-DB ( http://www.string-db.org, [22]).

**Figure 1:**
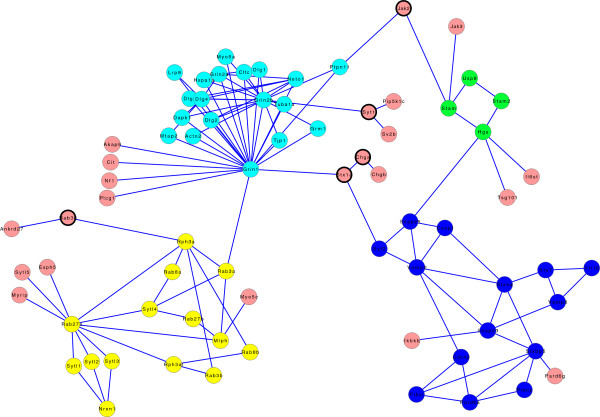
**The "smallPPI" network.** Each color other than pink denotes a cluster found with MCODE (see Data description). Pink nodes do not belong to any cluster. Note that the pink nodes with a thick line are not part of any cluster though they are connected with more than one edge.

As a starting point for the first network, we used the protein *Snap25* to search for proteins that are connected to it in string-DB. We extended this network further with indirectly connected proteins, until we obtained a network with 73 nodes and 116 edges (see Figure 1). The network has one connected component, which means, that each node is reachable from each other node. We refer to this network as the "smallPPI".

The second network was generated using a set of 62 proteins from Figure one in [23]. Each of these proteins was searched in string-DB, and networks were built containing this protein and its closest interactants. The networks were exported for each of the 62 proteins and then combined. The resultant dataset consists of 282 nodes and 501 edges. It has 18 connected components and is named "largePPI". The process of creating the networks is described in detail in the Additional file 1.

We first searched for complexes in these two networks using the software cytoscape ( http://www.cytoscape.org) with help of the plugin clusterViz ( http://chianti.ucsd.edu/cyto_web/plugins/displayplugininfo.php?name=ClusterViz) and using the MCODE method by [24]. This method finds groups of nodes with higher average edge–to–node ratios within the group than to nodes outside, and it is suitable for deriving protein complexes from graphs. These clusters were used as a reference for the complex prediction in the IP data later on. We simulated IP results from the two networks with an algorithm from [9]. This algorithm has the following biological basis:

During the biochemical isolation of the protein complex, some of the true protein interactions will likely break, resulting in isolation of a population of partial protein complexes. It is a logical and biochemically sound presumption that in an IP experiment, the probability to be pulled out is highest for the target itself (granted that the antibody works for the intended antigen), then high for its direct interactants and lower for the indirectly connected proteins. Thus, antibodies that target different complex subunits are expected to pull out slightly different protein subsets from the complex, which leads to redistribution of measured abundances for each protein.

**Figure 2:**
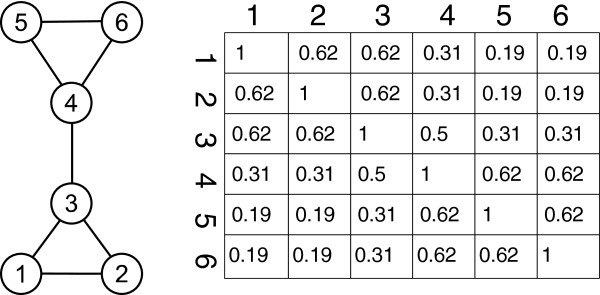
**Example graph and the corresponding connection probability table.** For a triangle like (1,2,3), the probability for each pair of nodes to stay connected is $\frac{5}{8}\approx 0.62$, as 3 edges lead to 2^3^ = 8 possible configurations of remaining edges where 5 of them enable at least one direct or indirect connection between 2 nodes. Image based on [9].

Kim et al. presented the following method to simulate this effect on graphs [9], where an unweighted undirected graph describes a protein network, nodes represent proteins, and edges represent interactions. First, they let a certain node in a graph to be the IP target protein, and each edge to have a breaking probability of 0.5 when the antibody pulls on the target. The probability for each node in the graph to stay indirectly or directly connected to the target is calculated, whereas the value for the target itself is set to 1, as it has the highest likelihood to be pulled out. Sets of nodes with many inner edges are likely to stay connected when one node of the set is the target. This corresponds to a high common abundance in the real IP experiment, where closely connected proteins stay together when the antibody pulls on one of them. Two only indirectly connected nodes are less likely to stay connected when one of them is the target, which leads to a low abundance value for the other one. The steps are repeated for each node to create a symmetric node by node matrix as shown in Figure 2. Finally, low values are removed from the matrix to make it sparse, -as typical IP results usually are-, with about 15% of non-zero values. In real studies, experimenters normally select the antibodies against a set of proteins that they consider being relevant. We simulate this through selecting only a subset of columns in the matrix for complex prediction instead of the whole dataset.

### Methods

#### The original 3N algorithm

**Figure 3:**
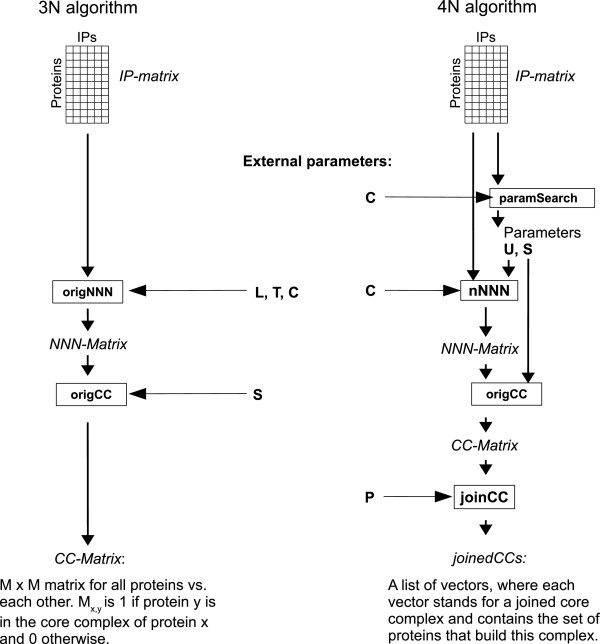
**Schematic overview of the 3N and the 4N algorithm.** The scheme contains all steps for the 3N and the 4N algorithm. The pseudocode for each of the steps is given in the Additional file 1.

This paragraph contains a brief description of the 3N algorithm, details can be found in the Additional file 1. An overview of the steps is given in Figure 3, left side. The 3N (Near Neighbor Network) algorithm by [17] was developed to detect minimal endogenous modules (MEMOs), which are stable complexes of proteins that always appear together in IP experiments. MEMOs are the smallest relevant units of interest and constitute the building blocks of larger core complexes and their isoforms.

3N defines the near neighbor network for a certain protein as the set of proteins that appear reciprocally or together, and have correlated abundance values with this protein across the different-antibody samples where it has its highest abundance. The correlation of two proteins is defined in terms of the cosine-distance between their rows *U*,*V* in the IP-matrix as *arccos*(*U* ∗ *V*/||*U*|| × ||*V*||) like explained in [25] and it has to be below a certain threshold. In the first step ("origNNN"), a NNN is calculated for each protein. These NNNs are used to infer the core complexes in the "origCC"-step. They represent sets of proteins that are very frequently pulled out together, regardless of the protein that binds to the antibody. A set of proteins, for which the NNN of nearly each member contains all other members of the set is a minimal core complex.

Although the original 3N algorithm performs well on the kind of data described in [18], it has some limitations. It has four external parameters that were manually optimized, and all of them influence the result (see Table 1). This is a strong limitation for generic applications of the algorithm, as the original parameter setup may not work as well and needs to be optimized for different datasets.

**Table 1:** Overview of the parameters for 3N and 4N

	**External for 3N**	**External for 4N**	**Affects totalnumber ofproteins incomplexes**	**Description**
L	Yes		Yes	Length of topList
T	Yes		Yes	Co-occurrence thresholdinfluencing param.
C	Yes	Yes	Yes	Cosinus-distance threshold
S	Yes	No	Yes	Jaccard coefficient threshold for building CCs
U		No	Yes	Jacc. coeff. threshold forbuilding NNNs
P		Yes	No	Jaccard coefficient threshold for joining CCs

External parameters are those that need to be adjusted by the user. The non-external parameters of 4N are set automatically by the algorithm.

To calculate the near neighbor network for one protein, 3N considers only the samples where this protein has its highest abundance values (i.e., the so-called Top list). This can lead to a loss of proteins when the length of the Top list (parameter L) is too short. A set of close proteins is only detected as core complexes when all their NNNs contain the complete protein set. Less closely connected complexes easily become predicted incompletely. A threshold of 65 for the cosine-distance (parameter *C*) is relatively weak for separating proteins that do not belong together, as two random vectors of only positive values are more likely to have a cosine-distance < 65 than one between 65 and 90. The 3N algorithm was not published as software implementation by the authors, which makes it impossible for others to immediately apply it to their own IP data.

#### The 4N algorithm

The aforementioned limitations motivated us to create the 4N algorithm based on the 3N idea. We removed the idea of sample ranking and used the binary Jaccard coefficient [26] for the protein co-occurrence in the step of NNN calculation. For a pair of proteins, this coefficient is the number of common occurrences divided by the number of samples where at least one of the proteins occurs. We automated the optimization of the most important parameters and added a step for joining overlapping complexes. For an overview of the algorithm, see Figure 3, right side.

Only two external parameters remain that need to be optimized manually for different datasets. One of them is the cosine-distance threshold parameter (*C*). In our analysis it is set to 40 such that the algorithm is able to keep proteins in one cluster that build an unbranched chain in the network. The value was determined experimentally using the two artificial datasets. This parameter has an influence on the absolute number of proteins in NNNs. The other external parameter (*P*), which is used to decide which core complexes should be joined finally, does not influence the total number of proteins that are put into complexes. Below we give an overview of 4N; a detailed description can be found in the Additional file 1.

##### 

**Inferring near neighbor networks and core complexes** We want to preserve all proteins that co-occur with at least one other protein in at least one sample. The Jaccard coefficient threshold for building the NNNs (*U*) is set to 0.01 initially to assign all proteins that fulfil this requirement to a near neighbor network. After this, *U* is set to the highest possible value, where each of the proteins still remains in at least one NNN. The cosine-distance threshold (*C*) is set to 40. The core complexes are calculated from the NNNs with the highest possible value for the Parameter *S* (Jaccard coefficient threshold for building CCs), for which all proteins occur in at least one core complex. Result of this step is set of core complex proteins for each protein in the dataset. These sets of proteins overlap highly, especially when they are built with a low *S*. For this reason, we added a step to join the core complexes (step "joinCC") based on the relative number of shared proteins.

##### 

**Joining core complexes** We define the overlap between two complexes as the relative number of proteins in the smaller complex that occurs in the larger one. When the threshold for joining core complexes (*P*) is smallest, all core complexes that share at least one protein are joined, which leads to completely distinct sets of joined core complexes. They represent groups of core complexes that interact within but not across the groups. Higher thresholds create smaller sets of partly overlapping complexes that stand for core complex isoforms, a value of 1 creates sets that represent MEMOs. Here, two complexes are only joined when the larger one contains all proteins of the smaller one.

**Figure 4:**
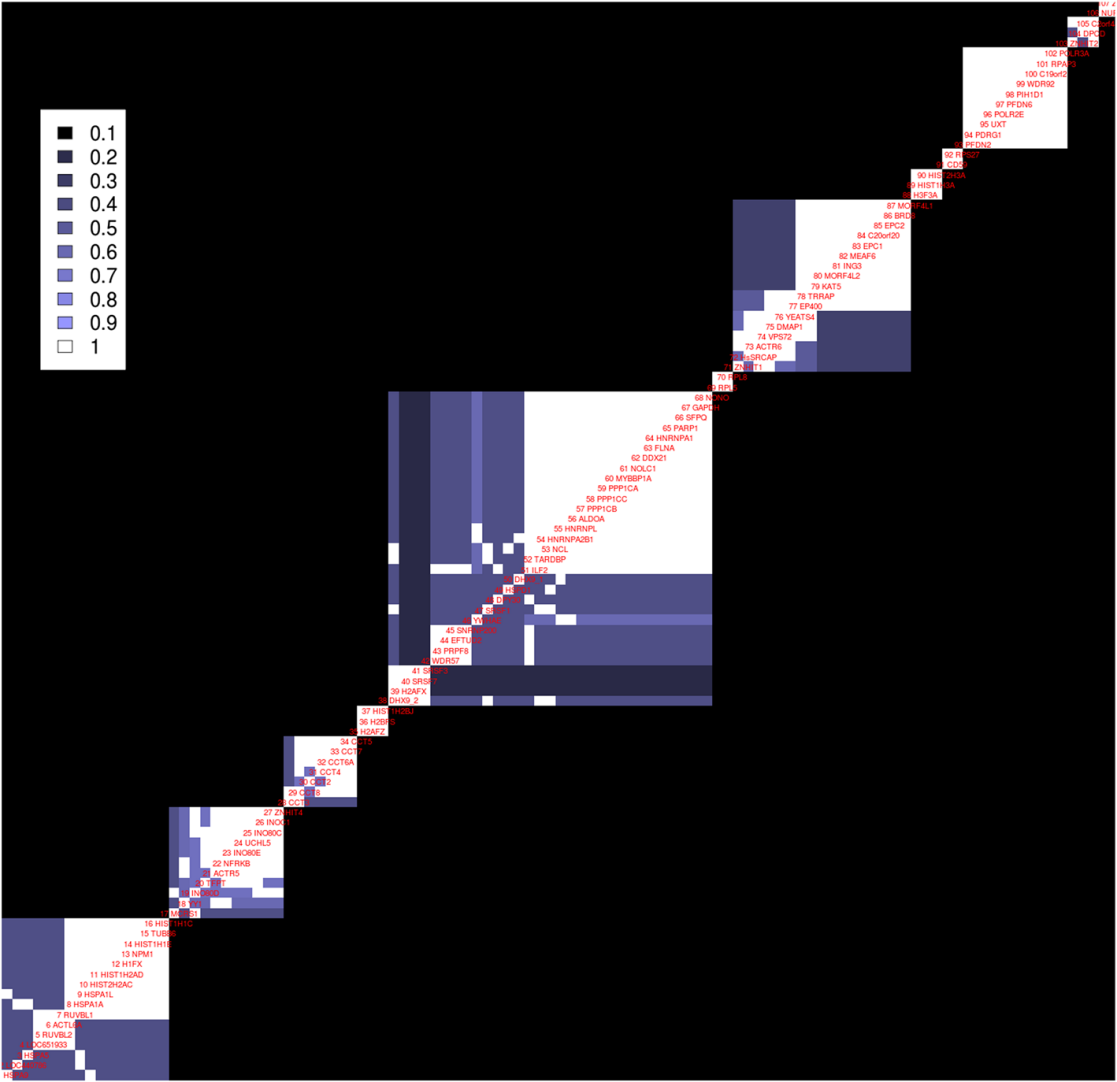
**Core complex plot for the "Tip49a/b" dataset.** Darker colors symbolize core complexes that were joined with a lower parameter *P* in the "joinCC"-step, brighter colors stand for higher *P*. 107 proteins were assigned to 12 non-overlapping sets of core complexes when *P* was 0.01. Higher *P* show the structure of cluster overlaps and reveal core complex isoforms. White solid squares show MEMOs. Explanations for all complexes can be found in the Additional file 1.

Different values for *P* in a range from 0 to 1 are used to create a core complex plot alike to the one in Figure 4. It is based on a matrix all proteins vs. each other. Each matrix cell is set to 0 at the beginning. A first joining–step is applied to the core complexes with the smallest *P*, and the matrix cells for proteins that are in the same joined core complex become 1, shown by the large non-overlapping squares in Figure 4. The joining step is repeated iteratively with higher *P* and the matrix cells for proteins that are still in the same core complex are incremented in each step. A standard hierarchical clustering with Euclidean distance is applied to each of the large squares to display a heatmap of the inner structure and the overlap of the joined core complexes. The image shows the MEMOs as well as the core complex isoforms and can be used for visualisation of the complex assignments for each protein. It also shows whether the automatic value for *U* is high enough to distinguish between the complexes. A too low *U* leads to one very large square in the plot with large white areas, see the plots for "Tip49a/b" in the Additional file 1 as example. The value must be set higher in this case. For a correctly set *U*, the plot can be used to set *P* to decide whether the results should contain larger, less overlapping complexes or smaller dense complexes that share proteins. The output of 4N for one specific set of parameters *U*,*C*,*S*,*P* is a list of predicted complexes. Each list element contains the names of the proteins that are in the complex.

#### Validation

The reference complexes for the three real datasets ("Tip49a/b", "malovIP" and "malovIP_subset") are those mentioned in the corresponding paragraphs in the Data description section. The reference clusters for the simulated data ("smallPPI", "largePPI") are those that were found by MCODE in the equivalent networks.

The threshold for joining the core complexes was selected for each dataset by examining the corresponding core complex plots and set to 0.5 for the artificial datasets, 0.6 for "Tip49" and to 0.85 for the tests on the "malovIP" datasets. The complex prediction quality was tested by comparing the 4N results with the reference clusters using the method by Brohée and van Helden [27].

Both the given and the predicted clusters can be seen as sets of sets. Let *G* be the set of given clusters, *P* the set of predicted ones. Each given cluster *g* ∈ *G* is seen as a set of proteins, likewise each predicted cluster *p* ∈ *P*. The scoring method uses the Jaccard coefficients for each combination of *p* and *g* as |*p* ∩ *g*|/|*p* ∪ *g*| and sets them in relation to the sizes and number of the real and predicted complexes. A complete description of the method can be found in [27]. The method calculates sensitivity, positive predictive value and as square root of the product of both, accuracy as quality measure. In addition, it gives the separation value as measure for how many predicted complexes represent one reference complex. This value is important because the accuracy does not decrease when a prediction method produces a too large amount of clusters that contain proteins from the reference complexes, as just the best overlapping cluster for each reference complex is taken into account.

A perfect complex prediction yields 1 for the accuracy score which means, that all reference complexes are completely covered by predicted complexes and the predicted complexes only contain proteins of their corresponding reference complex. The separation score is 1 when each reference complex is covered by exactly one predicted complex.

### Results

**Figure 5:**
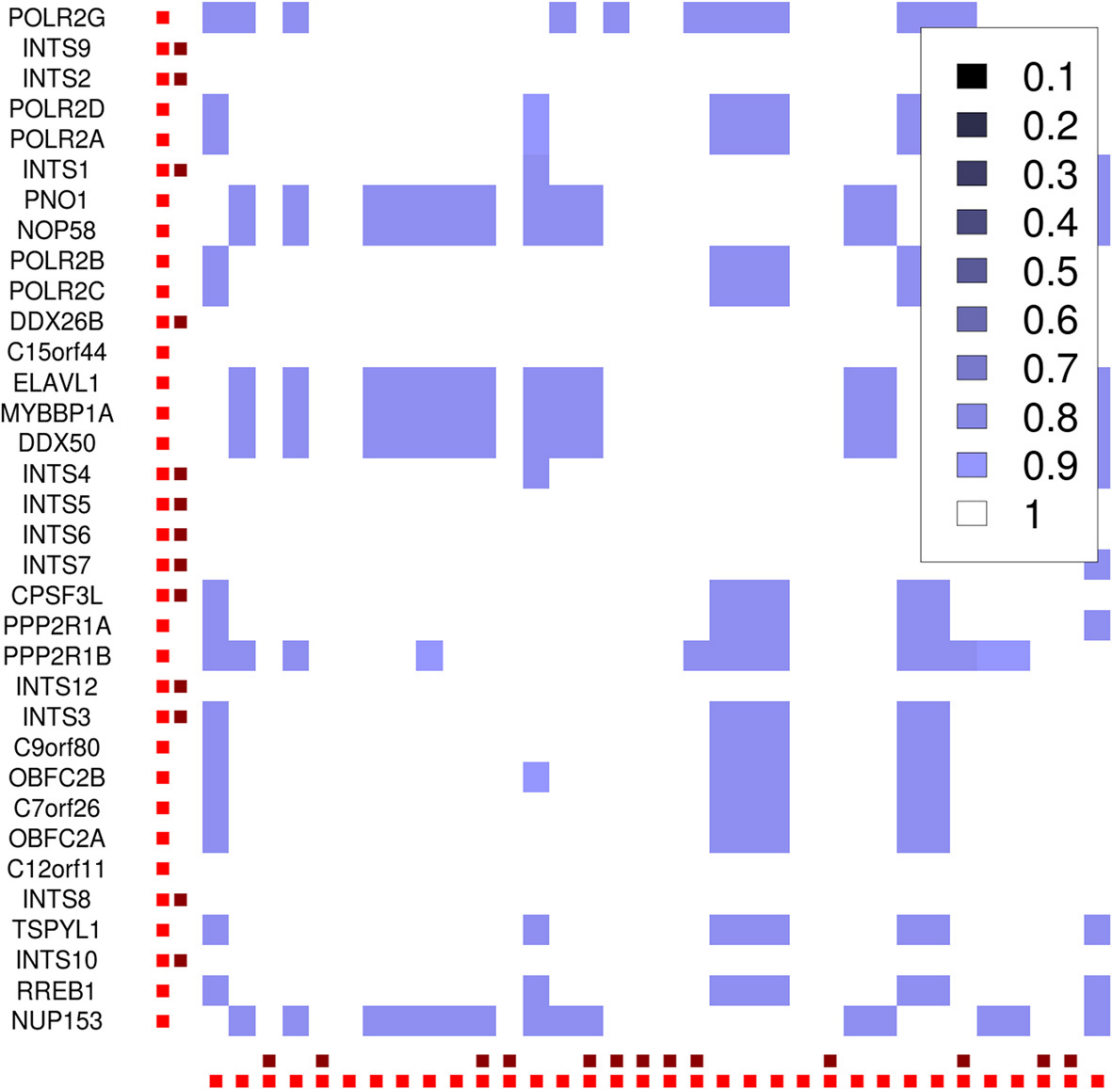
**Detail that shows the reference complex INT.** Dark red dots mark proteins that are in the reference-complex, the bright red dots the proteins in the complex predicted by 4N. The proteins from the complexes POL and INT are known from the literature to be close to each other and were put in one complex by 4N. A Figure that shows the close connections between POL and INT is in the Additional file 1.

**Figure 6:**
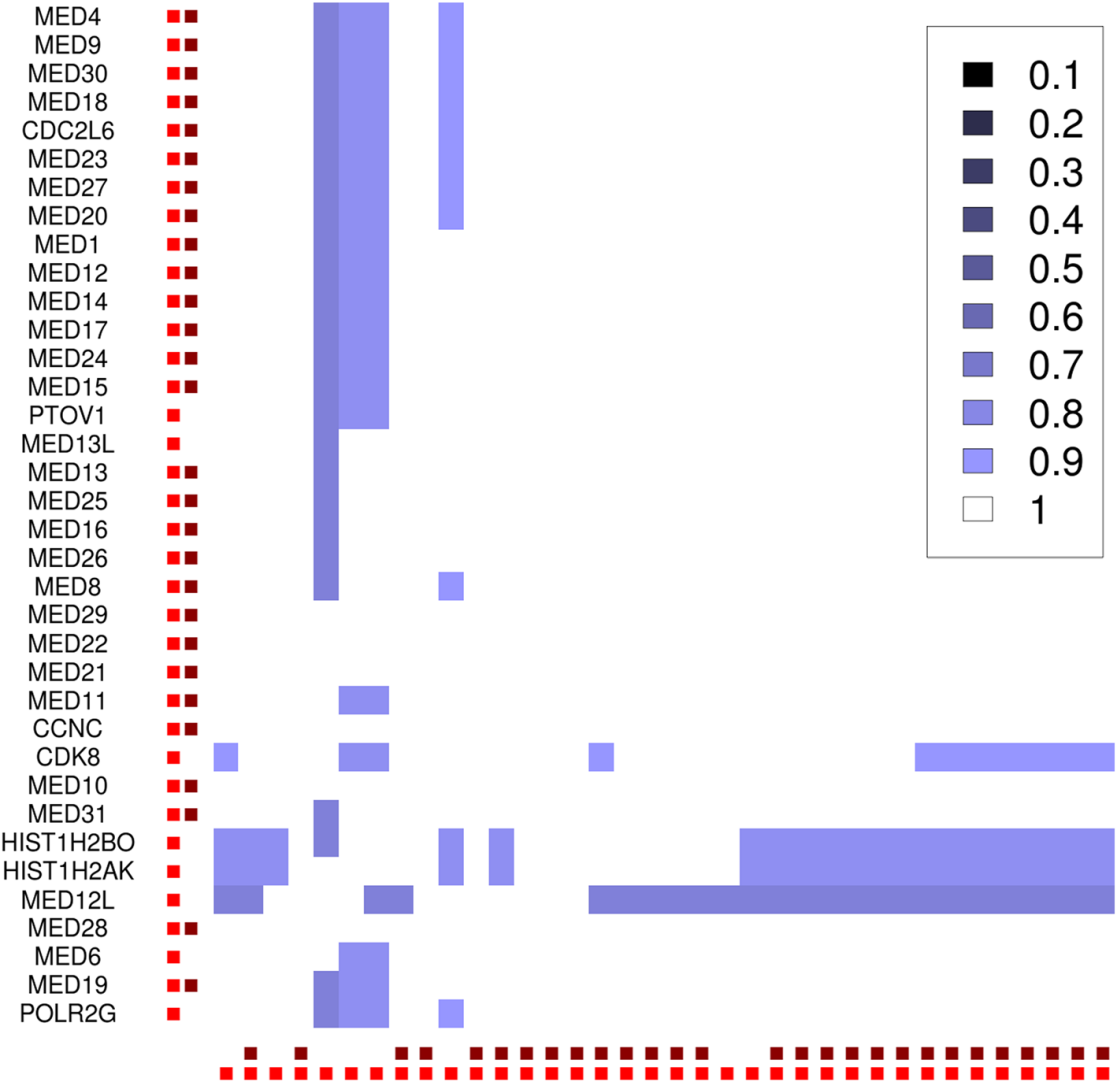
**Detail from the core complex plot for "malovIP" that shows MED.** White represents the MEMO, blue the core complex isoforms. The dark red dots at the axes mark proteins that are in the reference-complex, bright red dots the proteins in the complex predicted by 4N.

The two real datasets "malovIP" and "malovIP_subset" were analyzed and the joining-parameter (*P*) was set to 0.85 after examining the core complex plot. The predicted complexes were compared to the 10 reference complexes. On both datasets, the same scores were reached as all relevant proteins are in the "malovIP_subset" and the additional number of proteins in the "malovIP" dataset did not influence the prediction. Figures 5 and 6 show details from the core complex plot for two predicted reference complexes, the whole image and an explanation for all complexes are in the Additional file 1.

Some of the reference complexes, e.g., "POLII" and "INT", are known from the literature to be larger than the MEMOs shown in the original 3N publication [17] because the number of proteins assigned to the complexes varies from source to source. A search on string-DB for all participating proteins and their close interactors shows their neighborhood. The string-DB network can be found in the Additional file 1. In addition, other reference complexes are closely interacting with those complexes, see Figure three in [18]. Our predicted complexes were larger than the original reference complexes. For this reason, the PPV was 0.76 at a sensitivity of 0.83 and an accuracy of 0.8.

The analysis of "Tip49a/b" showed a core complex plot with one large core complex when the parameter *U* was automatically selected. The IP-matrix is very dense as the complexes interact closely, and many proteins are pulled out by the majority of the baits. A few proteins that are less closely connected cause the parameter *U* to be too low to distinguish between the close complexes, which can be seen in the core complex plot. We increased the parameter until the core-complex-plot showed more plausible complexes with a size of not more than 35 proteins. Core complexes were joined with a parameter *P* of 0.6. Different core complex plots for several *U* and the final plot including an explanation for all reference complexes can be found in the Additional file 1. The five largest complexes were predicted completely, but two of them are separated into two predicted complexes. Some small complexes with only 2 or 3 proteins were predicted incompletely, especially when they only contained bait proteins. The proteins that are not in reference complexes did not disturb the complex prediction but some of them appear in predicted complexes such as PPPase 1. The accuracy was 0.77 with a sensitivity of 0.67 and a PPV of 0.87. The separation was 0.49 which shows that 4N is assuming slightly too many complexes.

**Figure 7:**
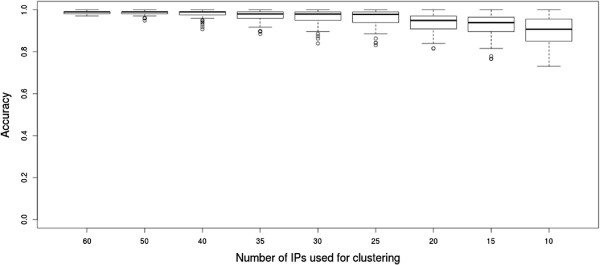
**Result overview for the "smallPPI" network.** Accuracy for 100 experiments each on 9 different numbers of samples.

The "smallPPI" IP dataset was processed first using all 73 columns as IPs. The accuracy was 0.99. A value of 1 was not reached, because 4N misclassified one single protein. We applied 4N 100 times each on random sample subsets with 60,50,40,35,30,25,20,15 and 10 IPs (see Figure 7). The average accuracy was around 0.99 for the experiments on 60 IPs and remained still above 0.89 for 10 IPs. The separation score ranges from 0.98 to 0.85. 4N performs as well as biclust on all tested samples and is about 100 times faster. In addition, the separation score for 4N is generally higher than for biclust in all experiments because biclust predicts a larger amount of complexes. Details on the comparison with biclust (runtime, scores and stability) can be found in the Additional file 1.

**Figure 8:**
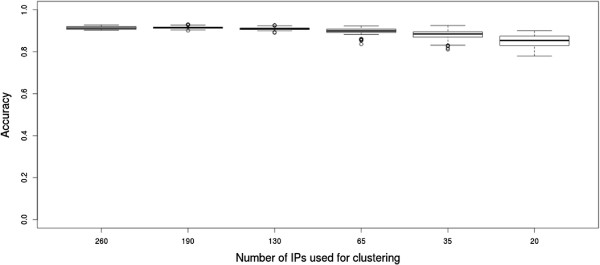
**Result overview for the "largePPI" network.** Accuracy for 100 experiments each on 6 different sample numbers.

The "largePPI" dataset was processed analogously, using all columns first. The accuracy was 0.91. The experiments were repeated on random subsets with 260,190,130,65,35 and 20 IPs (see Figure 8). The accuracy remained above 0.9 for the first 3 tests and was falling slightly for the second three tests, down to 0.85 when 20 samples were used for clustering. We also tried biclust on "largePPI" but the method terminates without giving an informative error message.

### Discussion and conclusion

Sensitivity and PPV of 4N highly depend on the selected parameters. Low *U* lead to a high sensitivity and low PPV, high *U* to the opposite. At a correctly set *U*, most of the complex proteins are found as complex proteins and assigned to the right cluster. In datasets where every protein has a high co-occurrence to at least one other one, this happens at the automatically set *U*. The parameter P has a smaller influence on the result especially when *U* is set correctly. A Figure in the Additional file 1 visualizes the effect on the Tip49a/b dataset. It is important to check different core complex plots to see the behavior when 4N creates one large cluster in the automatic setting.

We have set *U* in the experiment on "Tip49a/b" high enough to get at most 35 proteins per complex in the core complex plot. This value was selected because the size of the largest reference complex we had in any of our datasets was 35. The joining threshold was then selected high enough to preserve the information of closely connected subcomplexes within the large joined complexes.

For a biologist 4N might be more intuitive than algorithms that are based on probabilistic models such as biclust. As 4N does not need to be executed multiple times (which is necessary for probabilistic methods), it is very fast and can process very large IP datasets. The 4N analysis on the "malovIP"-dataset with its approx. 3200 IPs and 11500 proteins took approximately one day (Intel core 2 duo, 2 × 2.8 GHz), the analysis of "Tip49a/b" can be performed in seconds.

4N predicts the reference complexes in all tested datasets with high accuracy. The algorithm allows an immediate insight into the general density of an IP-dataset, the MEMOs, the core complex isoforms and the complex-complex interactions with the core complex plot. When this plot is seen as result, only one parameter that influences the result (the cosine-distance, *C*) remains instead of four parameters as in the original 3N algorithm. The immediate feedback given by the core complex plot allows assessment of the result and a manual adaptation of the parameters if necessary. 4N can process datasets of different density from dense to sparse. It is easy to install and execute as only a basic R-installation and one extra library is needed as minimum requirement. The method can efficiently cluster IP data and suggest protein complex compositions.

## Competing interests

The authors declare that they have no competing interests.

## Authors’ contributions

JK implemented the software and performed the calculations. In the joint writing of the paper IvM focused on the mathematical background, AM and ABS concentrated on the biology and HH and AKS coordinated the whole project. All authors read and approved the final manuscript.

## Supplementary Material

Additional file 1 Supplementary material. Click here for file
