# Supplementary material for: Inferring protein–protein interaction complexes from immunoprecipitation data
Source: BMC Res Notes. 2013 Nov 15;6:468. doi: 10.1186/1756-0500-6-468 (PMC3874675; doi:10.1186/1756-0500-6-468)

Inferring protein-protein interaction complexes from immunoprecipitation data

Supplementary Material

Contents

1 **Details on creating the artificial datasets** 2

2 **Schematic comparison between the original 3N and the new 4N algorithm** 3

3 **Description of the 3N algorithm** 4

4 **Description of the 4N algorithm** 5

5 **Runtime comparison for 4N and biclust** 7

6 **Cluster quality comparison of 4N and biclust** 8

7 **Influence of the parameters U and P on the behavior of 4N** 11

8 **Core complex plots for the Tip49a/b dataset** 12

9 **Final core complexes plot for the Tip49a/b dataset** 14

10 **Explanation for the Tip49a/b reference complexes** 15

11 **Core complex plot for the malovIP dataset** 16

12 **Explanation for the malovIP-reference complexes** 17

13 **The complexes POL and INT as PPI network from String-DB** 18

1 Details on creating the artificial datasets

smallPPI

We searched for the protein *Snap25* in the mus musculus database of stringDB. This protein takes part in the neuronal presynaptic PPI network. A network with this protein and the 20 most confident proteins around it was built with the stringDB website. Only interactions based on experiments and co-occurrence were used and the general confidence threshold was set to 0.15. We centralized the network to the node *stxbp5* and let the database extend the network by the max. 50 most confident proteins around this node. A few more nodes appeared. The procedure was resumed with the proteins *stx1a, rab3a, syt1, apbp* and *rab27a* until we gained a network with 73 nodes and 116 edges. A Figure of smallPPI can be found in the Paper.

largePPI

largePPI was generated using a set of 62 important proteins from the main Figure in Chua et al., 2010. Each of the proteins was searched on stringDB with the same parameters like the first network, and the max. 10 most confident nodes around it were displayed. This network was exported for each of the 62 proteins. Finally, we combined all networks and removed double nodes and edges. The result consists of 282 nodes and 501 edges. It has 18 connected components. A connected component in a graph is a subset of nodes that are all reachable from each other. There is no path between two connected components.

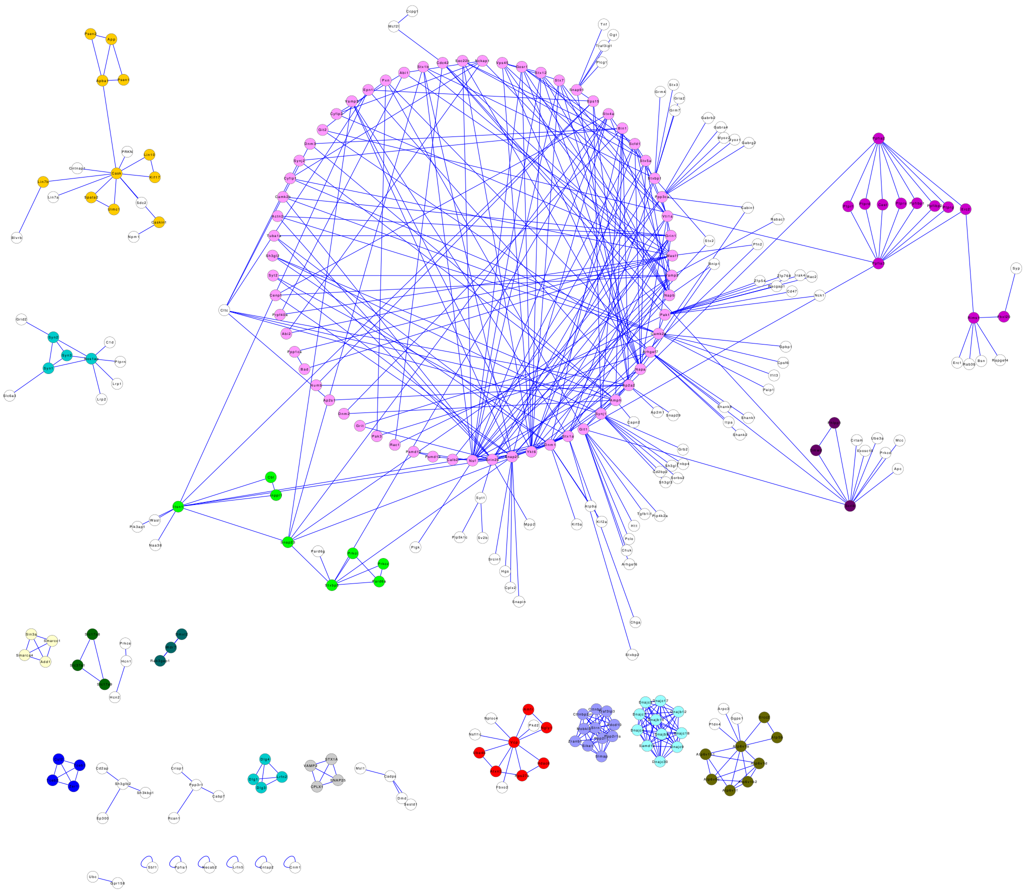

The largePPI network. Each color represents one of the complexes that were found by MCODE. White nodes are not assigned to any complex.

2 Schematic comparison between the original 3N and the new 4N algorithm

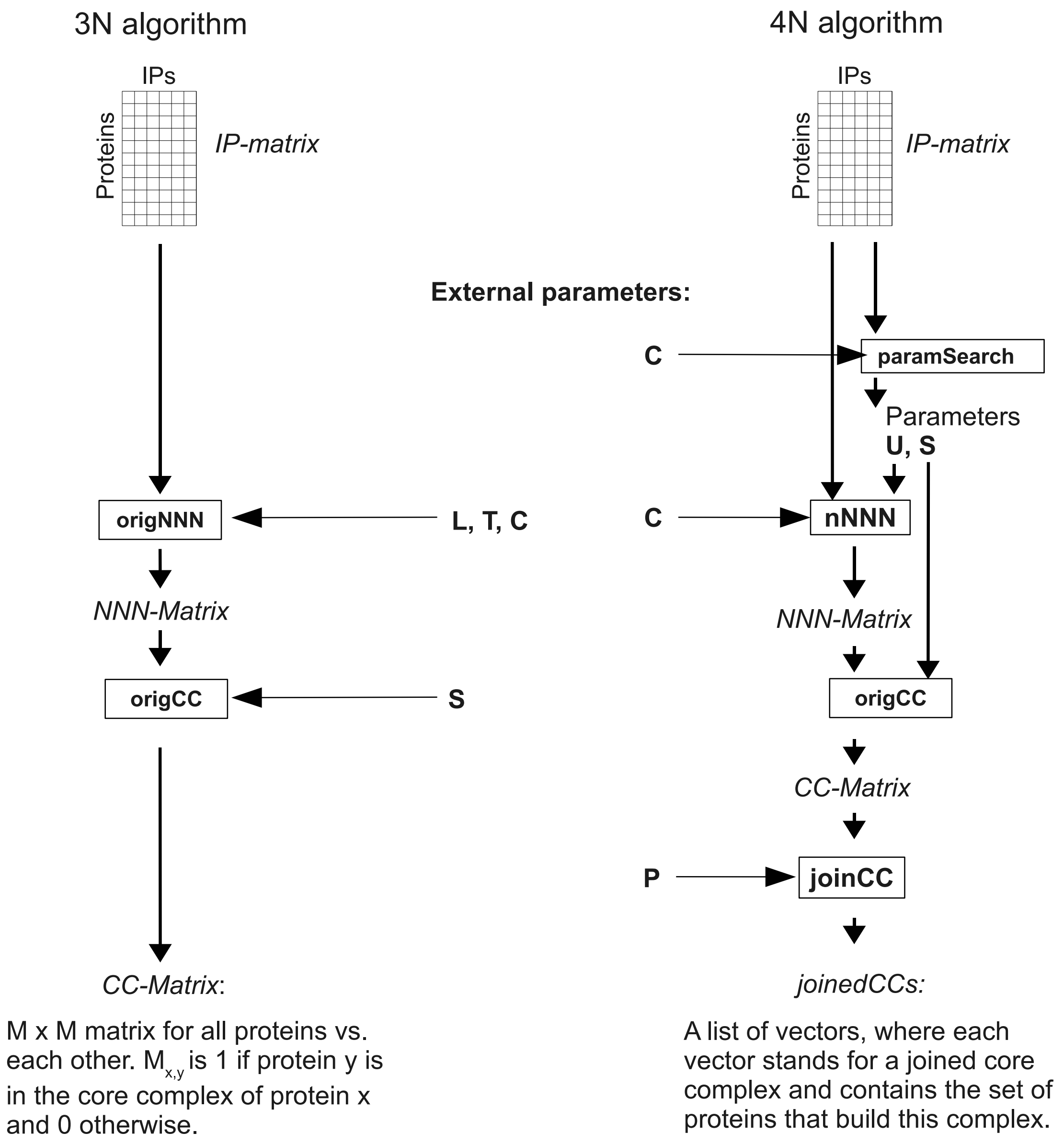

Overview of the parameters for both algorithms:

	External for 3N	External for 4N	Affects total number of proteins in complexes	Description
L	yes		yes	Length of topList
T	yes		yes	Co-occurrence threshold influencing param.
C	yes	yes	yes	Cosinus-distance threshold
S	yes	no	yes	Jaccard coefficient threshold for building CCs
U		no	yes	Jacc. coeff. threshold for building NNNs
P		yes	no	Jaccard coefficient threshold for joining CCs

3 Description of the 3N algorithm

IP-matrix:

M x N matrix where each row stands for a protein and each column for an IP-Bait. Each matrix cell $A_{m,n}$ contains the abundance value of a protein *m* in the IP-experiment with bait *n* or a 0 if the protein was not found in the experiment with this bait.

Original method of calculating the near neighbor network (origNNN)

Input: *IP-matrix*

External parameters, (values used in the original publication) :

L: Length of topList, (15)

T: Value to calculate the minimum protein co-occurrence threshold, (0.6)

C: Cosine-distance threshold, (65)

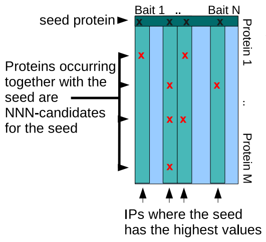

1 Create M x M matrix "*NNN*" with a 0 in each matrix cell.
2 Each protein is seed once. For the corresponding row of each seed in the IP-matrix:
  2.1 Select the **L** indicies of the baits where the abundance of the seed is highest and store these indicies in a list ("*topList*")
  2.2 Select the indices of all proteins that occur in the baits from the topList, and store their indicies in a "*NNN-candidate*" list
  2.3 Keep the indicies of those proteins in the NNN-candidate list that occur in at least $\mathbf{L}^{\mathbf{T}}$ baits from the *topList* and remove all other proteins
  2.4 Calculate the cosine-distance (see end of this page) between the seed protein and each protein in the *NNN-candidate* list.
  2.5 Keep the indicies of those proteins in the *NNN-candidate* list that have a cosine-distance ≤ **C** and remove all other proteins.
  2.6 Set NNN in the row of the seed for all column-indicies in *NNN-candidate* to 1

Output: *NNN*: M x M matrix for all proteins vs. each other. $NNN_{x,y}$ is 1 if protein y is in the near neighbor network of protein x and 0 otherwise.

Original method of calculating core complexes from the NNNs (origCC)

Input: *NNN-matrix*

External parameters, (values used in the original publication) :

S: core complex jaccard coefficient threshold parameter, (1)

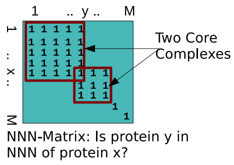

1 Create a M x M matrix "*CC*" with a 0 in each matrix cell
2 Each protein is seed once. For the corresponding row in *NNN* of each seed protein:
  2.1 Select the column indicies of all proteins that are in the nearest neighbor network of the seed (all column indicies where *NNN* is 1 in the row of the seed) and store them in a "*CC-candidate*" list
  2.2 Calculate the Jaccard coefficient (see end of this page) between the seed protein and each protein from the *CC-candidate* list
  2.3 Keep the proteins in the *CC-candidate* list that have a Jaccard coefficient ≥ **S** and remove all other proteins
  2.4 Set CC in the row of the seed for all column-indicies in the *CC-candidate* list to 1

Output: *CC*: M x M matrix for all proteins vs. each other. $CC_{x,y}$ is 1 if protein y is in the core complex of protein x and 0 otherwise.

Cosine-distance between the seed and a protein x in the *IP-matrix*:
Let **V1** contain the abundance values of the seed in all columns from *IP-matrix* that are in *topList*. Let **V2** contain the abundance values of protein x in the columns from the same topList.
cosine-distance(seed,x) = arccos(**V1 • V2** / ||**V1**|| **x** ||**V2**||).

Jaccard coefficient between the seed and a protein x:
Let **J1** be the column indicies where *NNN* is 1 in the row of the seed protein. Let **J2** contain the column indicies where *NNN* is 1 in the row of protein x.
Jaccard coefficient(seed,x) = |**J1** ∩ **J2**| / |**J1** U **J2**|.

4 Description of the 4N algorithm

New method of calculating the Near Neighbor Network (nNNN)

External parameters:

U: co-occurrence threshold

C: Cosine-distance threshold

Input: IP-matrix

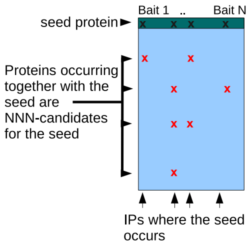

1 Create a M x M matrix "*NNN*" with a 0 in each matrix cell
2 Each protein is seed once. For one seed protein:
 2.1 Select the indicies of all baits where the seed occurs and store these indicies in a "*occurrenceList*"
 2.2 Select the indicies of the proteins that occur in the baits from the *occurrenceList*, and store these indicies in a "*NNN-candidate*" list
 2.3 Calculate the Jaccard coefficient (see end of this page) between each protein in the *NNN-candidate* list and the seed protein
 2.4 Keep the indicies of those proteins in the *NNN-candidate* list that have a Jaccard coefficient ≥ **U** and remove the other proteins
 2.5 Calculate the cosine-distance between the seed protein and each protein in the *NNN-candidate* list
 2.6 Keep the indicies of those proteins in the *NNN-candidate* list that have a cosine-distance ≤ **C**
 2.7 Set NNN in the row of the seed to 1 for all column-indicies in *NNN-candidate*

Output: *NNN*: M x M matrix for all proteins vs. each other. $NNN_{x,y}$ is 1 if protein y is in the near neighbor network of protein x and 0 otherwise.

Jaccard coefficient between the seed and a protein x:
Let **K1** be the set of column indicies in the IP-matrix where the seed occurs, **K2** the column indicies where x occurs.
Jaccard coefficient(seed,x) = |**K1** ∩ **K2**| / |**K1** U **K2**|.

Cosine-distance between the seed and a protein x in the *IP-matrix*:
Let **V1** contain the abundance values of the seed in all columns from *IP-matrix* that are in ***K1*** ∩ ***K2*** and **V2** the abundance values of protein x in the same columns.
cosine-distance(seed,x) = arccos(**V1** • **V2** / ||**V1**|| x ||**V2**||)
If |**K1** ∩ **K2**| == 1 then
 If Jaccard coefficient(seed,x) > 0.5 then cosine-distance(seed,x) = 0
 else cosine-distance(seed,x) = 90

The if-clause is necessary because when two proteins share only one sample, it is unlikely that they are in the same complex when their Jaccard coefficient is low. However, is this sample the only sample that contains that proteins at all, they are more likely to be in the same complex.

Note: A low sample number increases the chance that two indirectly interacting proteins are in just one (the same) sample. This leads to a reduced sensitivity. The Jaccard coefficient is less meaningful here and highly influenced by the sample selection.

Method of searching the ideal parameters for nNNN (paramSearch)

External parameters:

C: Cosine-distance threshold, (40)

Input: *IP-matrix* of only relevant proteins

1 Run nNNN on the *rIP-matrix* with **U** = 0.01, **C** = 40 and store the result in "*NNN_current*"
2 Count the number of proteins that are in at least one near neighbor network
3 Search the highest possible value for **U** in a range between 0.01 and 1 where running nNNN with **C** = 40, **U** leads to the same number of counted proteins like in step 2
4 Run origCC with **S** = 0.01 on the *NNN* from step 3 that was produced with this highest **U** and store the result in "*CC_current*"
5 Count the number of proteins that are in at least one core complex
6 Search the highest possible value for **S** in a range between 0.01 and 1 where running origCC with **S** on this *NNN* leads to the same number of counted proteins like in step 5
7 the values **U** from step 3 and **S** from step 6 are the output

Output: ***U***, **S:** They are the highest possible parameters for running nNNN and origCC with having all proteins from the rIP-matrix in core complexes.

Method of joining core complexes (joinCC)

External parameters:

P: Core complex jaccard coefficient treshold, (0.5)

Input: *CC* (output from origCC)

Note: One row x in *CC* represents the core complex that belongs to the protein x.
1 Create an upper triangle matrix "*toJoin*" which rows and columns represents all core complexes vs. each other
2 For each combination of two row indicies *a*,*b* from *CC*:
2.1 Let **A** be a vector with the column indicies of *CC* that contain a 1 in the row *a*, **B** the same for the row *b*
2.2 If the ratio between the number of indicies that are in both vectors **A,B** and the total number of indicies in the shorter vector is at least **P**, set $toJoin_{a,b}$ to 1
3 See *toJoin* as a adjacency matrix of a *graph**
3 Create a list of vectors "*joinedCCs*"
4 For each connected component** in *graph*:
4.1 Select all core complexes (rows from *CC*) from the current connected component
4.2 Select all column indicies where *CC* has at least one 1 in one of the selected rows and append this vector of column indicies to joinedCCs

Output: *joinedCCs*: A list of vectors, where each vector stands for a joined core complex and contains the set of proteins that build this complex.

* Let each core complex be a vertex in a graph and let $toJoin_{x,y}$ contain a 1 when an edge exists between vertex x and vertex y. *toJoin* is called the adjacency matrix of this graph.

** A connected component is a maximum set of vertices where each vertex can be reached directly or indirectly from each other vertex in the set.

5 Runtime comparison for 4N and biclust

The most time–consuming step of 4N is ”nNNN”. It is also the only step that benefits from using multiple processors in the MPI-version of 4N. Parallelization creates some overhead and is not recommended for datasets smaller than ”largePPI”.
The ”nNNN” is repeated for different values for U while searching for the best value for this parameter, which makes the runtime depending on the density of the IP dataset. Our experience shows that U is usually between 0.1 and 0.4, so ”nNNN” is executed between 2 and 12 times. Finding the best parameter for S is fast as calculating the core complexes does not consume much time. The step of joining the core complexes and creating the core complex plot is more time consuming again, as the joining-step is performed multiple times with different thresholds.

On small to medium size datasets of less than 400 proteins and IPs, 4N finishes within minutes and creates the core complex plot. The runtime is growing roughly linear to the data size. A runtime overview for our datasets is given in the following table. All tests were performed on a PC with Intel core 2 duo CPU (2 x 2.8 GHz) and 4 GB of memory. The table shows the runtime of 4N including the calculation of the core-complex plot in comparison to biclust.

Dataset	#Proteins x #IPs	Runtime of 4N	Runtime of biclust
smallPPI	73 x 73	10 sec.	20 min.
TIP49	126 x 35	45 sec.	25 min.
largePPI	281 x 282	165 sec.	N/A
malovIP_subset	3290 x 3311	approx. 6 hours	N/A
malovIP_full	11500 x 3311	approx. 25 hours	N/A

Runtime comparison of 4N and biclust based on the tests with different sample numbers on smallPPI. 4N's runtime was measured here without the core complex plot.

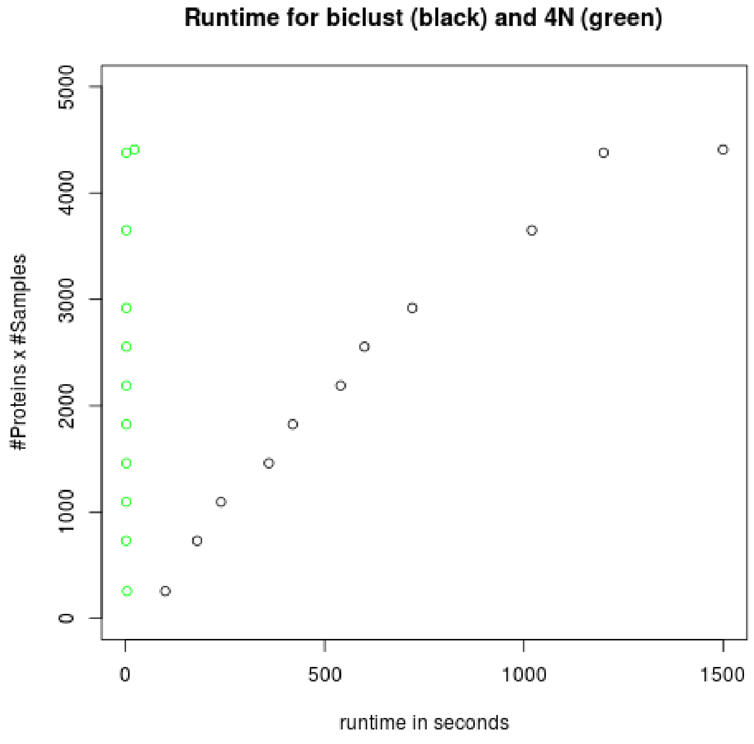

6 Cluster quality comparison of 4N and biclust

Three versions of smallPPI were used for the comparison between biclust and 4N, beginning with the original smallPPI dataset. A version of smallPPI with added gaussian noise was created for the second test. The third test was performed on a smallPPI version where every value > 0 was set to 1.

100 Runs on random subsets for each number of samples were performed with 4N and because of limited time, 20 runs with biclust. On this dataset, 4N and biclust are neither prone to noisy data nor to occurrence data.

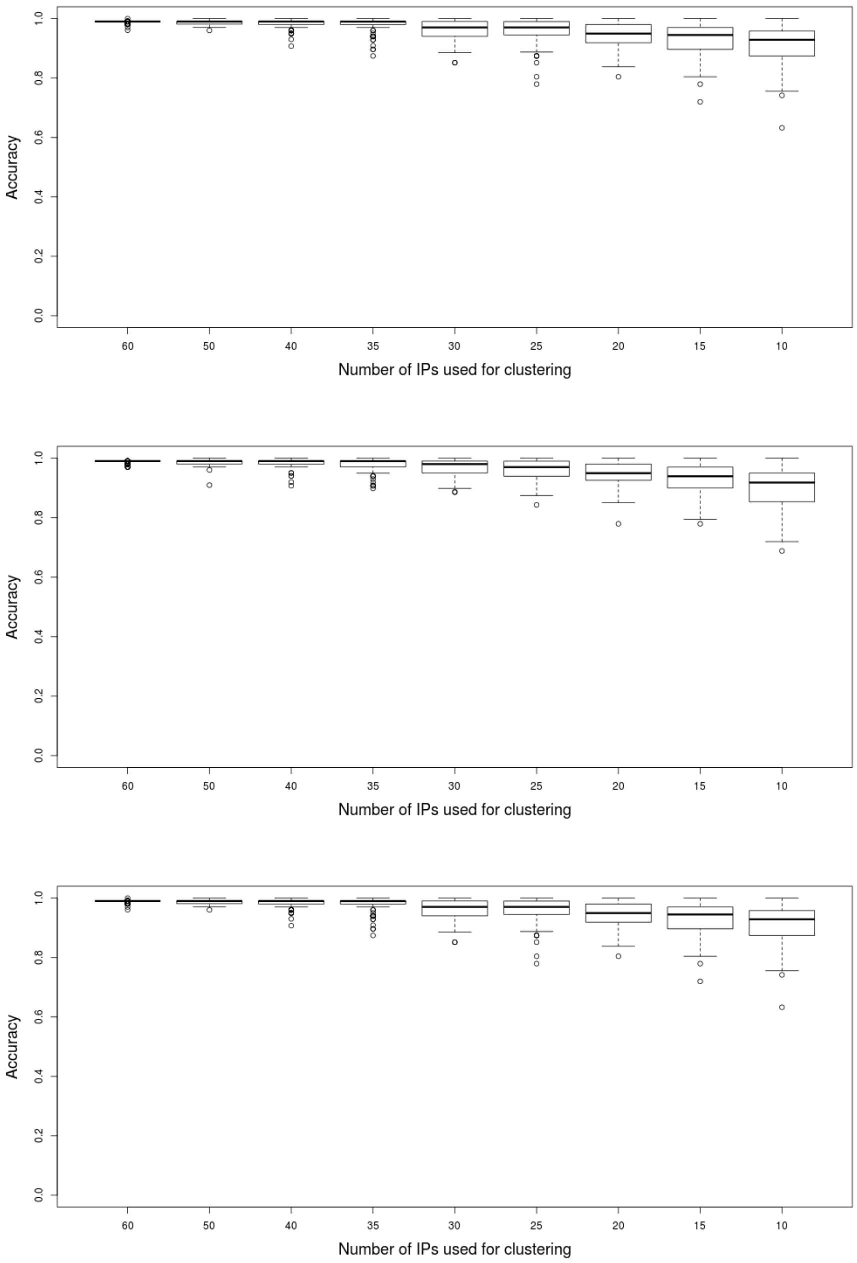

Figure 1: Top: 4N on original smallPPI, middle: 4N on data with added gaussian noise, bottom: 4N on occurrence (0/1) data

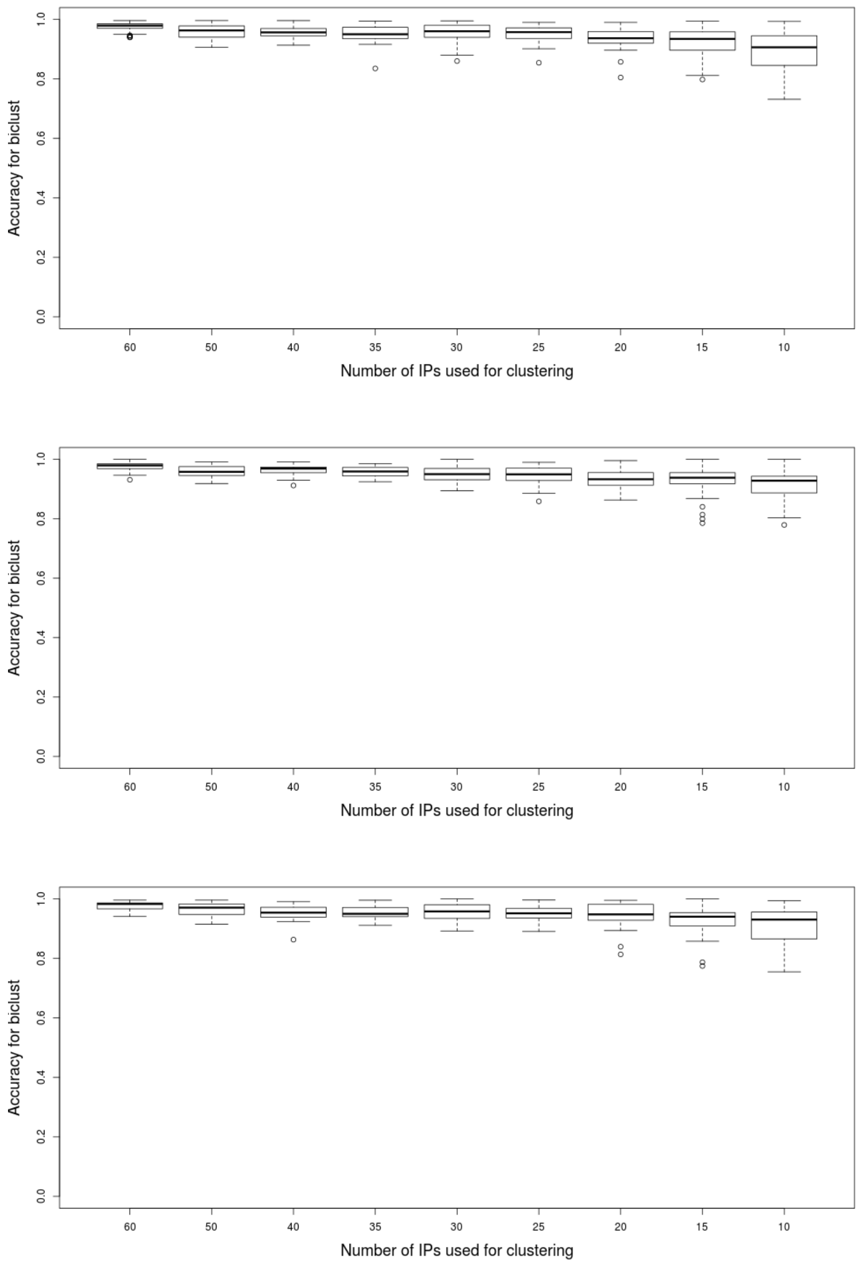

Figure 2: Top: biclust on original smallPPI, middle: biclust on data with added gaussian noise, bottom: biclust on occurrence (0/1) data

The boxplots show that 4N and biclust provide comparable results on all three datasets in terms of accuracy. The sensitivity and PPV (not shown here as Figure) is highly similar as well.

The comparison of the separation quality shows a different picture. Biclust is prone to assume too much highly overlapping complexes. Sensitivity and PPV are not influenced by that, as the scoring method chooses the best prediction of each reference complex for calculating the scores.

The separation of biclust is getting better on a low amount of samples because biclust clusters the samples first and then the proteins within the sample clusters. Less samples lead to less possible sample clusters. 4N clusters the prey proteins directly and creates a more realistic amount of protein clusters on this example.

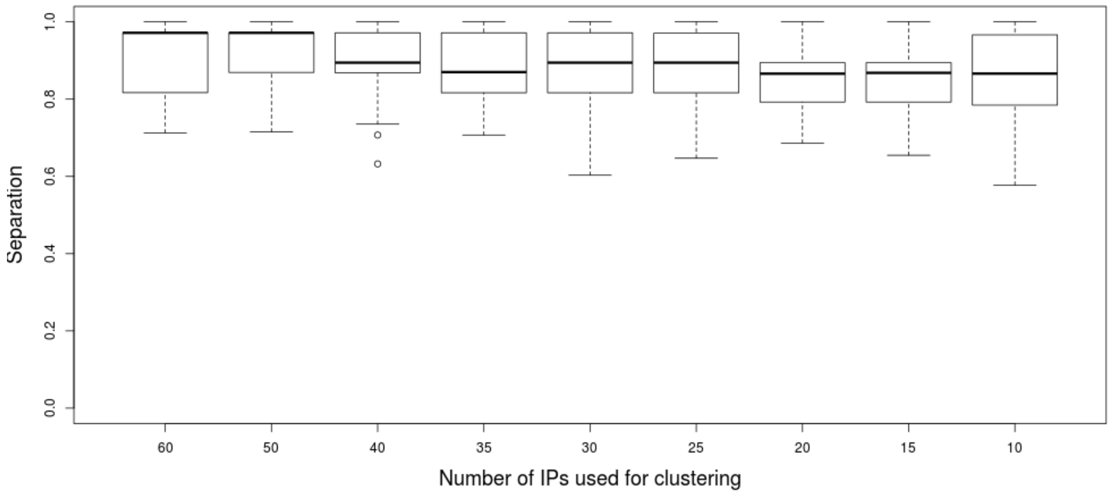

Figure 3: Separation for 4N on smallPPI

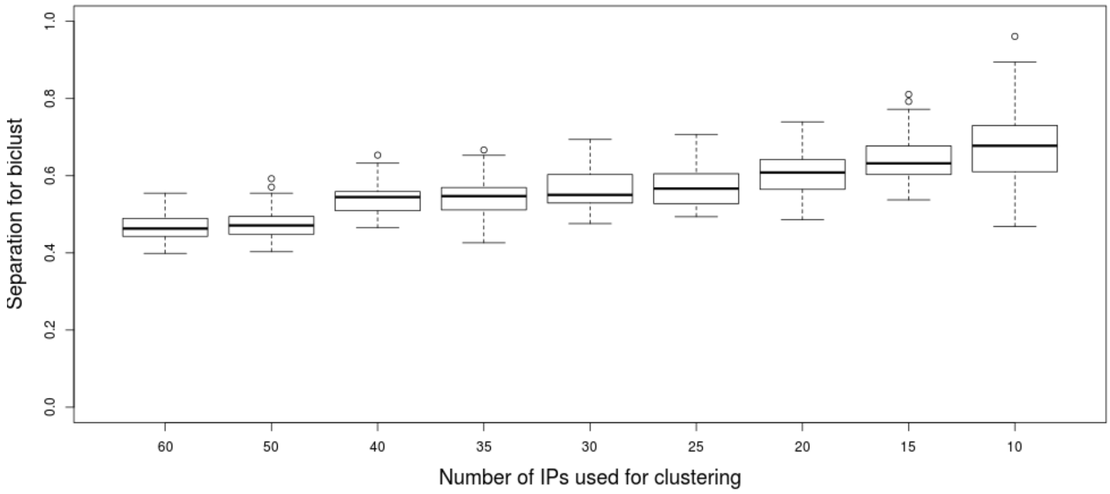

Figure 4: Separation for biclust on smallPPI

7 Influence of the parameters U and P on the behavior of 4N

The parameter U is a threshold for the jaccard coefficient for two proteins and therefore, decides how often they need to co-occur to be in a cluster. A low U of 0.01 assigns all proteins to a certain protein with any co-occurrence to it, a U of 1 would only assign proteins that always co-occur to a cluster.
The parameter S is the reciprocity threshold to build core complexes from the NNNs. It can be set manually too, but it should be kept at the automatically set value. The parameter P is the treshold for the jaccard coefficient between two core complexes and decides whether they should be joined. The core complex plot, created by 4N in its automatic setting, gives an impression on how closely connected the proteins in the dataset are. When U in the automatic setting is too low (for example, below 0.1), very large complexes appear which contain large white areas in the plot. This is an indicator that 4N cannot distinguish between the complexes in the dataset. U should be set higher in this case and core complexes should be created for the higher settings to see when 4N starts to separate the complexes.

The following Figure shows the influence of U and P on sensitivity, PPV and accuracy of 4N on the Tip49a/b dataset. It contains 100 experinemts. 10 different U from 0.1 to 1 were tried and for each U, 10 different P in the same range. S was selected automatically for each run. The plot shows that there is a large influence of P when U is set too low and just a small influence when U is set correctly. The sensitivity is high for too low U (logically) at a very low PPV and falling for higher U. Only a few complexes in IP-datasets have a perfect jaccard coefficient, which makes 4N too strict at very high U, leading to a bad sensitivity. Oh the other side, it is unlikely that two proteins that co-occur often do not belong to the same complex, which leads to the very high PPV for high U.

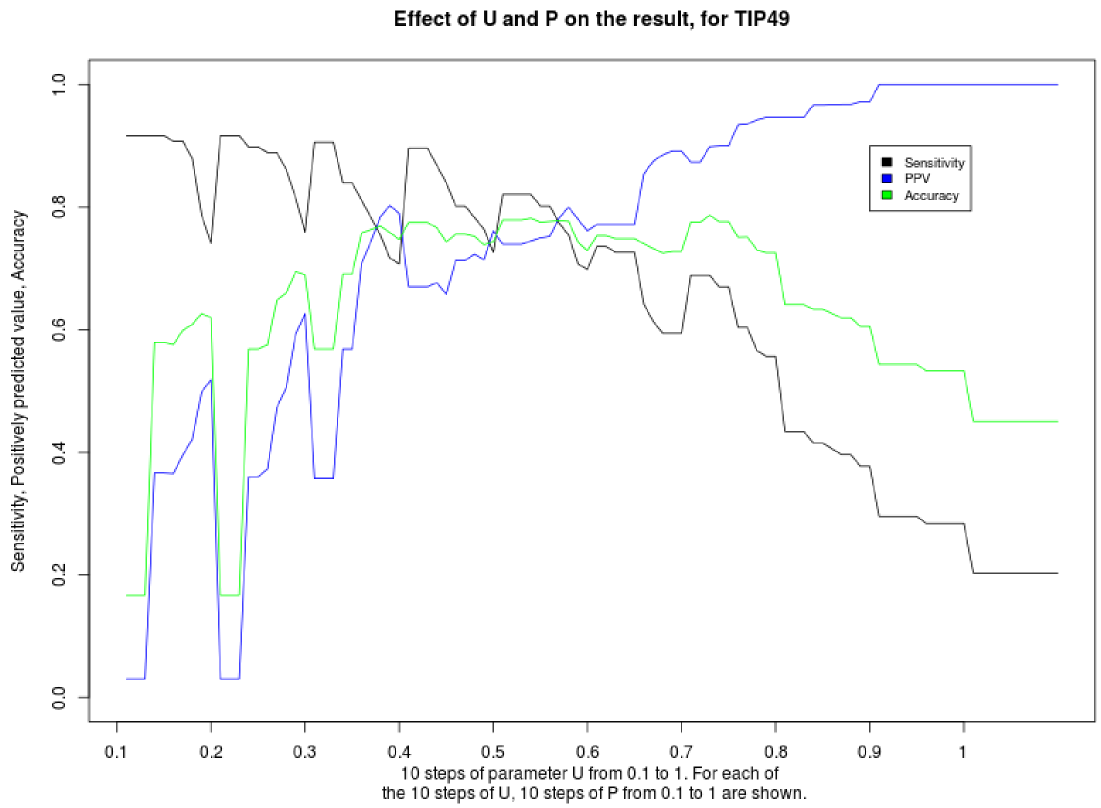

8 Core complex plots for the Tip49a/b dataset

Applying 4N with its automatic setup on the Tip49a/b dataset leads to a parameter U of 0.25 and to a core complex plot where almost all proteins are predicted to be in one complex. This is logical for a dataset of this high density. U was increased stepwise to see the effect on the cluster result.

For low U, one very large complex appears with large white areas. This shows that 4N does not distinguish between the single complexes in this low setup.

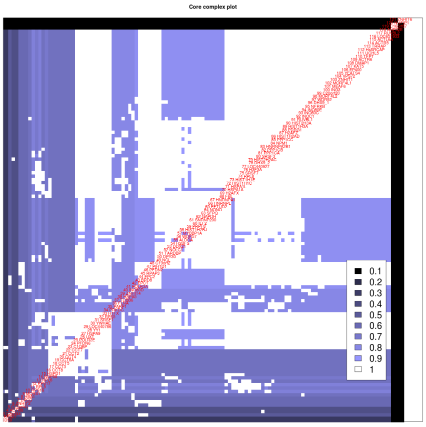

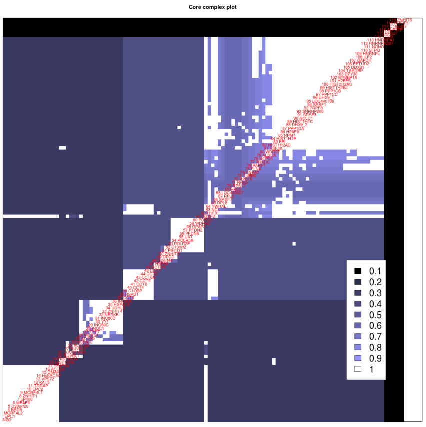

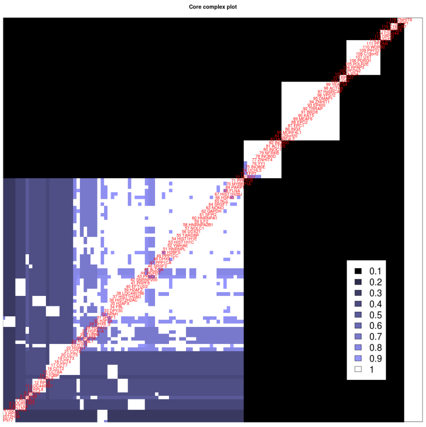

Figure 5: U = 0.2 U = 0.3 U = 0.4

Plots with higher U show more details. At a U of 0.6, no large white square remains but most of the proteins are still captured. This value was chosen for the final result.

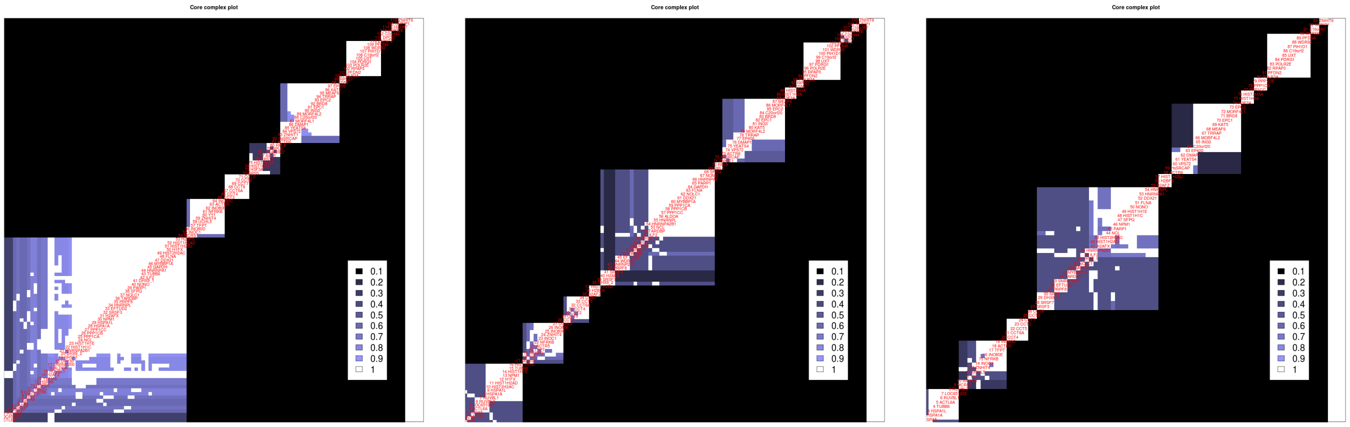

Figure 6: U = 0.5 U = 0.6 U = 0.7

At even higher (too high) U, more and more proteins are lost and less blue occurs in the plot. This shows that the parameter P does not influence the result anymore, because the remaining complexes do not overlap.

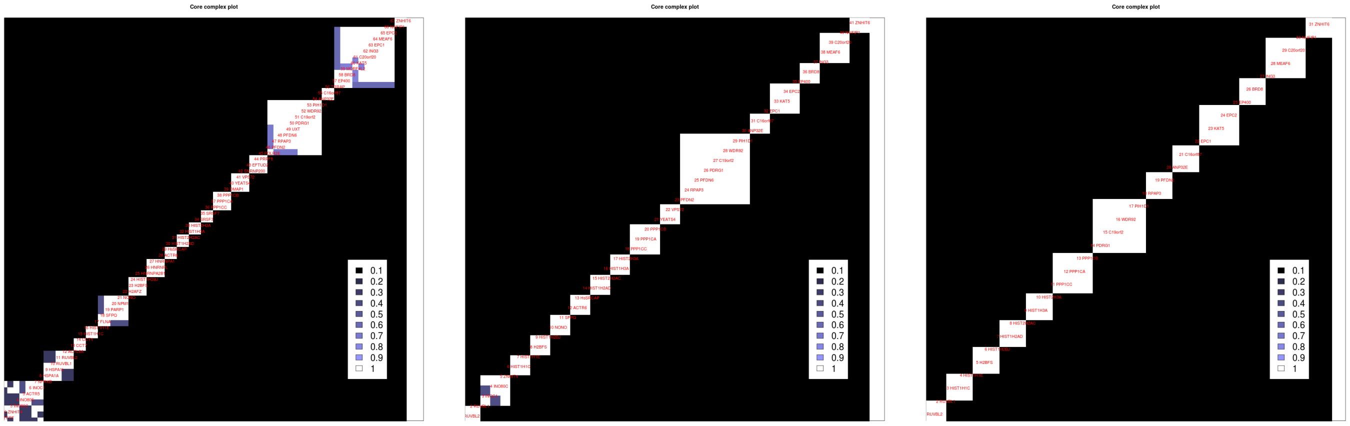

Figure 7: U = 0.8 U = 0.9 U = 0.95

9 Final core complexes plot for the Tip49a/b dataset

107 of the 125 proteins were put into core complexes during the analysis of TIP49a/b. Dark colors stand for a low parameter P for joining the core complexes, brighter colors for higher values.

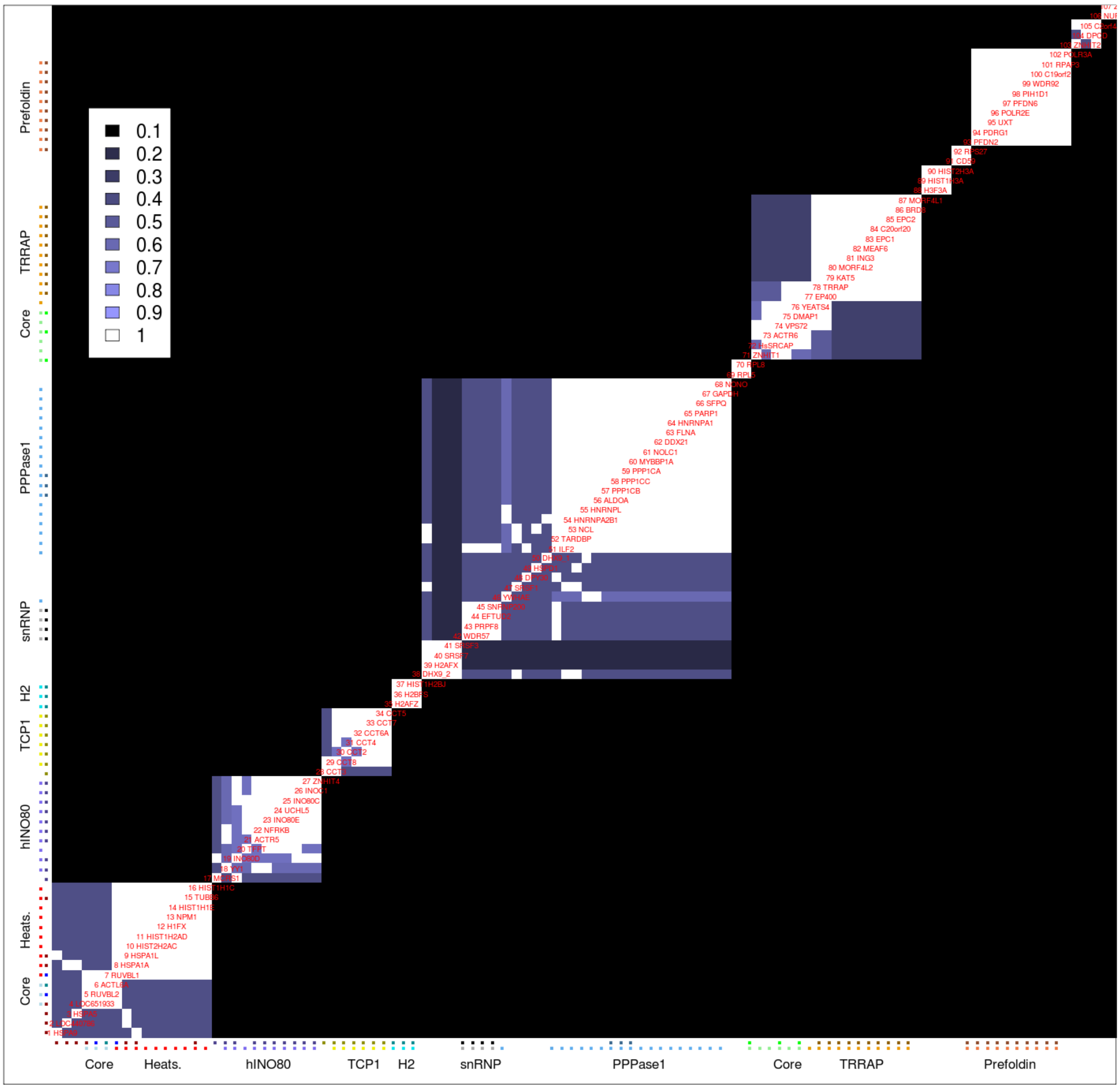

The darker colored lines of dots at the edges mark the positions of the 11 reference complexes from the Figure in Stukalov et al., 2012. The brighter dots beneath them mark the clusters predicted by 4N.

10 Explanation for the Tip49a/b reference complexes

The core complexes were joined with P=0.6. An accuracy of 0.77 was reached for this result.

Core

Two complexes are named with core in the publication by Stukalov et al.. The first complex with the proteins DMAP1, YWATS4, VPS72 and ZNHIT1, was predicted completely, the other one (RUVBL1 and RUVBL2) was predicted as larger complex that contains both proteins.

TRRAP

TRRAP was predicted completely.

U5 snRNP

This complex was also predicted completely.

Chaperonin TCP1

This complex appears as one complex in the core complex plot. When joining the core complexes with a threshold of 0.6, two proteins get split away from the complex to form an own one. The Jaccard-coefficient is 0.86 instead of one for this reason.

hINO80

This complex also appears as one complex in the core complex plot, but is split into two complexes in the joining step. The Jaccard-coefficient is 0.82.

H2 enriched

One protein of H2 is assigned to another complex than the other three. The Jaccard coefficient is 0.75.

SRCAP

The two proteins are assigned to different complexes. This complex is not marked in the core complex plot.

Prefoldin

Prefoldin was predicted completely.

Protein phosphatase 1

All three proteins were assigned to the same complex, but together with many other proteins.

Heatshock 70kDA enriched

This complex was predicted as three different, but overlapping complexes. Two of them share one protein (and were not joined because the overlap is below 0.6), the third also contains RUVBL1 and RUVBL2. The Jaccard coefficient is 0.29.

11 Core complex plot for the malovIP dataset

3311 of the 11485 proteins were put into core complexes during the analysis of the "malovIP" dataset. The Figure contains only the 408 proteins in core complexes that contain at least one protein from one of the reference complexes. The image is diagonally symmetric and shows all proteins vs. each other. Dark colors stand for a low parameter P for joining the core complexes, brighter colors for higher values. Most of the proteins are joined to one huge cluster that covers almost the whole image when P is set low. At values around 0.5, smaller and more stable core complexes start to be visible.

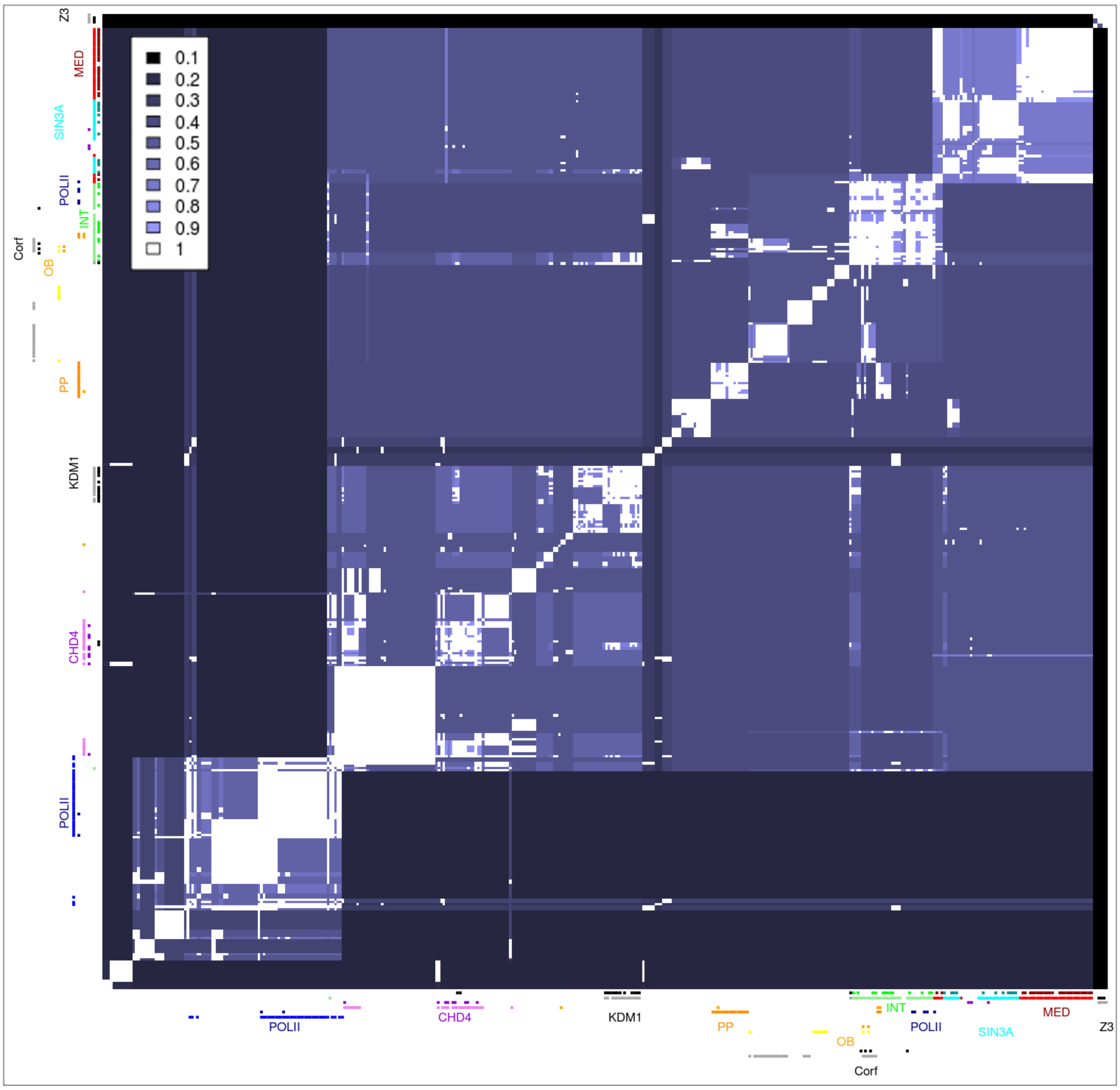

The darker colored lines of dots at the edges mark the positions of the 10 reference complexes from the Figures in Malovannaya et al., 2010. The brighter dots beneath them mark the clusters predicted by 4N.

12 Explanation for the malovIP-reference complexes

The core complexes were joined with P=0.85. The accuracy was 0.8.

INT

The INT-complex was predicted completely, but together with proteins from the complexes POLII, OB and Corf. Reason is the high co-occurrence of proteins from this complexes. The Jaccard coefficient between the reference and the prediction was 0.48.

POL

The POLII complex itself was predicted incompletely, with a coefficient of 0.29. The missing proteins were assigned to the INT-complex instead, which is known to be closely connected to the POL complex.

PPPase

Three of the five proteins were predicted correctly. The predicted complex contains further proteins that are known to be close to the PPPase-complex from the literature. The Jaccard coefficient was 0.75.

Corf

This reference complex, which consists of uncharacterized proteins, was predicted incompletely and together with other proteins. The Jaccard coefficient was 0.25.

Med

The MED-complex was predicted completely. Some of the proteins in the predicted complex were not in the reference complex though they had the prefix ”Med”. The coefficient was 0.97, because one of the proteins from the POL-complex was assigned to the MED-complex.

OB, Z3

Both complexes were found completely with a Jaccard coefficient of 1. Both predicted complexes contained further proteins that are known from the literature to be connected to OB and Z3 but that were not in any reference complex.

CHD4, SIN3A, KDM1

These three complexes share proteins and are also known to be closely connected.
CHD4 was predicted with a coefficient of 0.56 as several proteins of the reference complex were missed. The correctly detected proteins were combined with other proteins that were not in any reference cluster.
SIN3A was predicted with a Jaccard coefficient of 0.83, KDM1 with 0.71. Some proteins in the three predicted complexes were assigned to other complexes in this group.

13 The complexes POL and INT as PPI network from String-DB

As stated in our publication, the complexes POL and INT are closely interacting. To substantiate this, we searched for all proteins which were assigned by 4N to POL and INT as well as all proteins from the reference complexes, including neighbor proteins. The node colors symbolize whether a protein is part of POL / INT according to the reference / 4N. Orange nodes are neighbor nodes.

Yellow are the proteins that are assigned to INT by 4N but not by the reference, green are the ones that are assigned to INT by both 4N and reference. POLR2H and PLOR2E belong to the the POL complex in the reference and for 4N, but the blue proteins are assigned to POL by 4N as well. Red are proteins that are assigned to INT by 4N but belong to POL.

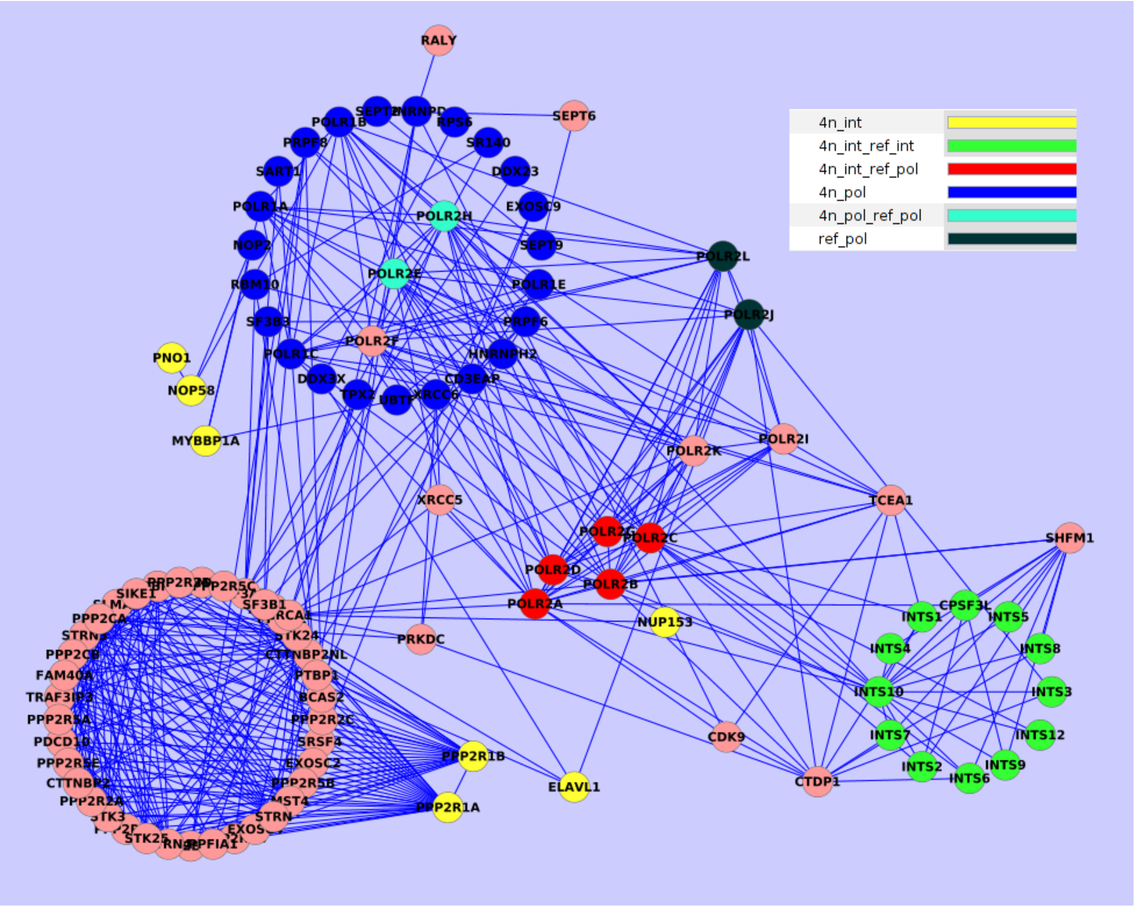

Supplement: Additional file 1 — Supplementary material. [file 1756-0500-6-468-S1.pdf]
